# First Experience of Treatment of Multiple Shrapnel Traumatic Pseudoaneurysms During the War in Ukraine Using Tegus Telemedical System

**DOI:** 10.1007/s00062-023-01319-6

**Published:** 2023-06-29

**Authors:** Dmytro Shchehlov, Mykola Vyval, Adnan H. Siddiqui, Rene Chapot, Oleksandr Pastushyn, Oleksandr Hnelytsia, Jens Fiehler, Vladimir Kalousek, Anna A. Kyselyova

**Affiliations:** 1 Research and Practical Centre for Endovascular Neuroradiology, the NAMS of Ukraine, Platon Mayboroda st., 32, Kyiv, Ukraine; 2 Department of Neurosurgery, University at Buffalo Jacobs School of Medicine and Biomedical Science, Buffalo, NY USA; 3 Department of Neurosurgery, Gates Vascular Institute, Buffalo, NY USA; 4 Department of Intracranial Endovascular Therapy, Alfried Krupp Hospital Essen, Essen, Germany; 5 Neurosurgical Department, Zhytomyr Regional Clinical Hospital, Zhytomyr, Ukraine; 6 Department of Diagnostic and Interventional Neuroradiology, University Medical Center Hamburg-Eppendorf, Martinistr. 52, 20246 Hamburg, Germany; 7 Department of Radiology, University Clinical Hospital Center Sestre Milosrdnice, Zagreb, Croatia

Treatment of cerebrovascular injuries in Ukrainian neurointerventional centers has faced numerous challenges in the past year and is still largely done on a case-by-case basis. When vessel walls are damaged, false aneurysms or pseudoaneurysms can occur, resulting in an encapsulated hematoma that communicates with the ruptured artery [1]. If left untreated, the mortality rate due to cerebral bleeding from untreated cerebral pseudoaneurysms can reach 50% [2, 3]. While the neurointerventional armamentarium offers many options for treating traumatic pseudoaneurysms, the best treatment strategy requires experienced interventionalists and advanced planning, with periprocedural reconsideration. Remote proctoring offers unique advantages for surgeons dealing with unfamiliar and complicated cases and has the potential to transform the current medical world into one where patient safety is not constrained by location. Additionally, telemedical proctoring can provide an opportunity for less experienced practitioners to learn from more experienced colleagues and receive immediate feedback on their performance [4].

A patient in the 20s was admitted to a regional military hospital in Ukraine in early March with multiple shrapnel injuries to the head, chest, and legs. The patient was initially comatose (GCS 9) and tetraplegic with two penetrating head wounds, one in the right frontal region (inlet hole from the shrapnel) and the other in the left occipital lobe (exit hole). After undergoing surgical debridement, the patient was transferred to the intensive care unit (ICU) for supportive care until improvement. Non-enhanced cranial computed tomography (CT) revealed bone fragments near the pericallosal artery (Fig. 1) and CT angiography confirmed both pericallosal artery injuries with two pseudoaneurysms on the right side and one on the left side (Fig. 2).

**Fig. 1:**
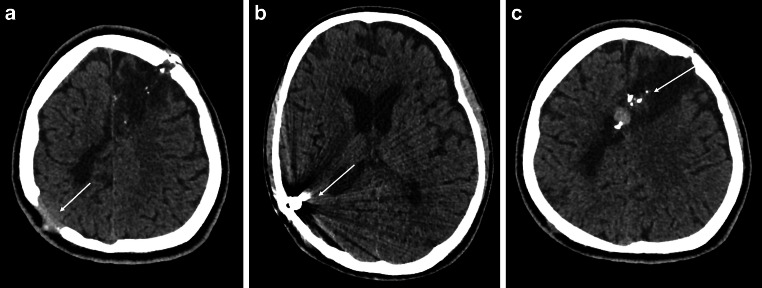
Non-enhanced cranial CT image in axial section of a diametrical shrapnel injury. **a** The entrance in the left frontal area, exit (*arrow*) in the right parietooccipital area with postcontusional parenchymal lesions. **b** Shrapnel near bone margins inside the brain parenchyma (*arrow*). **c** Bone fragments, that caused the injury of both ACA in the midline (*arrow*)

**Fig. 2:**
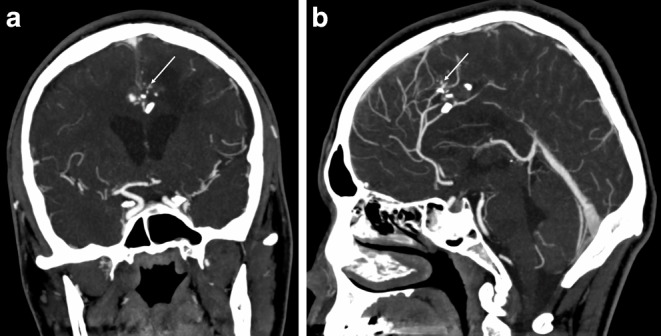
CT angiography of the cerebral arteries in coronal (**a**) and sagittal (**b**) views with traumatic aneurysms and bone remnants in the projection of the pericallosal arteries (*arrows*)

The clinical course was complicated by sepsis, but after 4 weeks, the patient’s consciousness improved (GCS 14) and movement in the right extremities was achieved, although the patient remained left-sided hemiplegic. In May, the patient was transferred to the center in Kyiv for additional diagnostic angiography (DSA) and pseudoaneurysm treatment. The DSA revealed one bigger right pericallosal (3 × 4.7 mm) traumatic aneurysm that was located distally, a smaller proximal one (0.5 × 0.5 mm) on the side branch (Fig. 3a), and a small (0.6 × 0.5 mm) left sided aneurysm (Fig. 3b). Subsequently the patient was transferred to the main military hospital where antibiotic treatment was continued, and endovascular treatment strategy was planned.

**Fig. 3:**
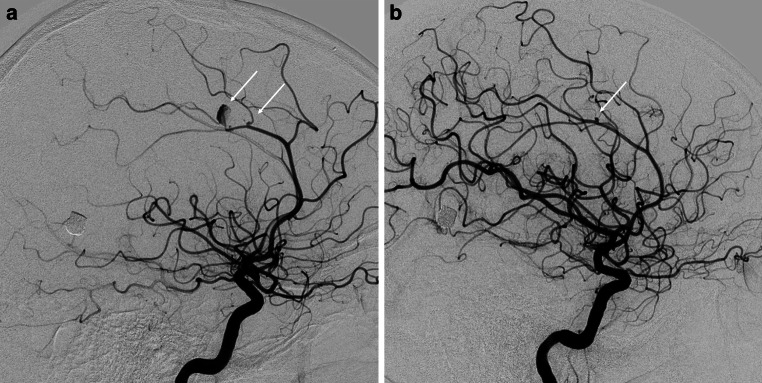
Digital subtraction angiography of the cerebral arteries with two traumatic aneurysms of the right pericallosal artery (*arrows*) (**a**). Small aneurysm of the left pericallosal artery (*arrow*) (**b**)

After online discussion with the contributing international experts, we made the initial treatment plan. In the first stage, we considered treatment of the right pericallosal artery after the balloon test occlusion, as reconstruction of pseudoaneurysms according to our and worldwide experience can seldomly be reached with stable occlusion, causing peri-interventional or postinterventional rupture [1]. In addition, we were not certain that flow diversion can lead to a good outcome considering the small diameter of the artery and traumatic stenosis on the level of the distal aneurysm of the right pericallosal artery. Therefore, we could not exclude the risks of stent occlusion and vessel rupture either; however, the unpredictable course of multiple traumatic pseudoaneurysms argued for the re-evaluation of this strategy during the procedure. All treatment options were considered, and international cooperation with Professor Fiehler (Department of Diagnostic and Interventional Neuroradiology, University Medical Center Hamburg-Eppendorf, Hamburg, Germany) was decisive. Thus, an interdisciplinary discussion in the ESMINT (The European Society of Minimally Invasive Neurological Therapy) community, as well as peri-interventional support of the telemedical system Tegus (Tegus Medical GmbH, Hamburg, Germany), were organized. Tegus offered a great opportunity for real-time collaboration, exchange, and guidance in this challenging case, being able to connect to other specialists from all over the world.

After 3 weeks of the initial angiography, experts scheduled an endovascular procedure which was accepted by all parties. During the peri-interventional angiography, it was found that the smaller right-sided aneurysm had increased considerably (measuring 2.3 × 2.4 mm), the larger one had decreased (measuring 4.5 × 2.5 mm), and the left-sided aneurysm remained unchanged (Fig. 4). A balloon occlusion test of the right pericallosal artery was performed, and good collateral filling was observed in the investigated area (Fig. 5), as well as the filling of the distal aneurysm. The parent artery was treated with distal to proximal coil embolization, and both aneurysms were excluded (Fig. 6). After the telemedical proctoring live case session, it was concluded that the initial plan was successfully conducted, and all experts’ considerations were taken into account during the intervention (Video).

**Fig. 4:**
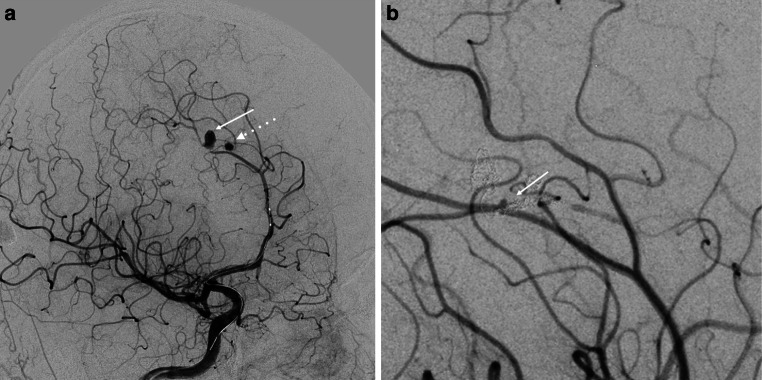
Peri-interventional digital subtraction angiography of the cerebral arteries revealed size changes in the right pericallosal aneurysms: increase of the one of the aneurysms (*dotted arrow*) and decrease of the other one (*arrow*) (**a**) and stability of the left aneurysm (*arrow*) (**b**)

**Fig. 5:**
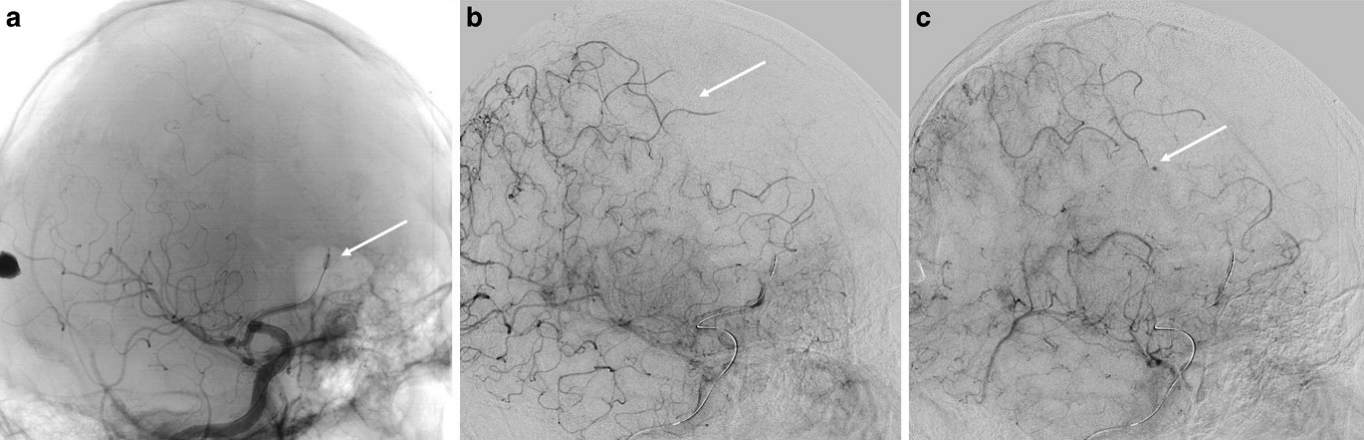
Peri-interventional imaging of the cerebral arteries. **a** Balloon-test occlusion of the right ACA (HyperForm 4 × 7 mm; EV3, Irvine, CA, USA). **b** Collateral crossflow between right ACA and MCA. **c** Aneurysm filling through collateral pathway

**Fig. 6:**
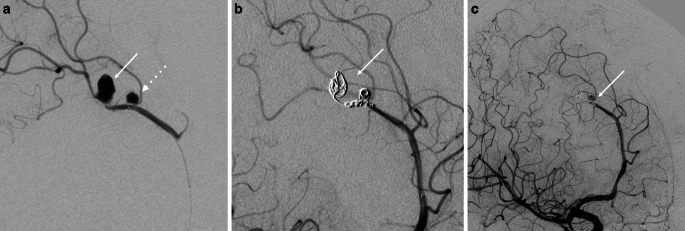
Microcatheter angiogram showing right pericallosal aneurysms (*arrow and dotted arrow*) (**a**). Coil embolization of the distal aneurysms (Cosmos 4 mm × 8 cm; Microvention, Aliso Viejo, CA, USA); second coil (Helical 2 mm × 8 cm; Microvention) was placed between distal and proximal aneurysms. Part of this coil was put in the proximal aneurysm. Embolization of the aneurysms was finished with Penumbra Smart Coils 1 mm × 2 cm and 1 mm × 3 cm (Penumbra, Alameda, CA, USA) (*arrow*) (**b**). Angiographic results after procedure with contrast stagnation in the proximal aneurysm (*arrow*) with adequate collateral filling (**c**)

After successfully securing the more bleeding-prone aneurysms on the right, the next stage involved planning the reconstruction of the left pericallosal artery using a stent. As the patient was stable and did not experience any new neurological decline, dual antiplatelet therapy with 100 mg aspirin and 75 mg clopidogrel was initiated 3 days after the procedure. Then 5 days later a stent (LEO Baby; Balt, Montmorency, France) was implanted in the left pericallosal artery at the level of the aneurysm, and the procedure was successfully completed (Fig. 7).

**Fig. 7:**
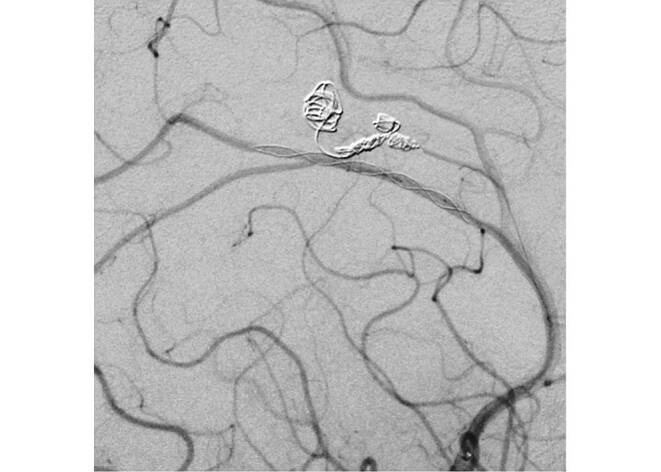
For the second session a LEO Baby (2 mm × 18 mm) stent (Balt, Montmorency, France) was successfully deployed on the level of the left pericallosal aneurysm with immediate result. Aneurysm is not visualized

## Outcome and Follow-up

After 6 months the patient showed moderate left-sided hemiparesis with a modified Rankin scale (mRS) score of 2, and is still undergoing neurorehabilitation. Clopidogrel was discontinued 6 months after the endovascular procedure. No additional cerebral infarction occurred after the treatment.

## Discussion

Traumatic distal pseudoaneurysms are a rare occurrence in neurointerventional practice, and treating them can be challenging, especially when they appear in multiple locations. Our data show that even a short deferment of the treatment of multiple traumatic pseudoaneurysms requires re-evaluation of treatment strategy during the procedure. Recent advances in interventional neuroradiology have provided numerous tools to address various types of congenital and acquired pathology; however, dealing with the multiplicity of traumatic aneurysms requires a non-trivial approach in decision making to balance the risks and benefits of simultaneously preserving blood flow and excluding aneurysms. Ideally, highly experienced neurointerventionalists should collaborate and provide guidance to achieve the best possible outcome. In this espect telemedicine plays a crucial role in providing this support.

## Conclusion

In times of war, there is a wide range of penetrating traumatic injuries to the head region, including those affecting the cerebral vessels. The utilization of telemedicine with remote proctoring can be a valuable additional option for neurointerventional practice during such cases. The main challenge during the treatment is to preserve blood flow and to exclude aneurysms at the same time, with a careful weighing up of the risks and benefits.

### Supplementary Information

Video: Treatment of the right-sided pericallosal aneurysms using Tegus telemedical system
